# Embodying well-being in research: key principles and praxis

**DOI:** 10.3389/feduc.2026.1836519

**Published:** 2026-06-25

**Authors:** Hadley Rahrig, Megan Lipsett, Adam B. David, Caroline G. Richter, Ya-Yun Chen, Em V. Adams, Caitlyn L. Wilson, Senegal Alfred Mabry, Vincent D. Lai, Kathrine Whitman, Grace O. Allison, Ellie P. Xu, Lindsey Ostermiller, Samantha De Leon Sautu, Claire Laubacher, Christine D. Wilson-Mendenhall

**Affiliations:** 1 Center for Healthy Minds, University of Wisconsin-Madison, Madison, WI, United States,; 2 Department of Psychology, University of Oregon, Eugene, OR, United States,; 3 Department of Psychological Sciences, University of Connecticut, Storrs, CT, United States,; 4 Department of Psychology, The University of Alabama at Birmingham, Birmingham, AL, United States,; 5 Psychology Department, Virginia Polytechnic Institute and State University, Blacksburg, VA, United States,; 6 Parks, Recreation, and Tourism Management, Clemson University, Clemson, NC, United States,; 7 Clinical Health Psychology, University of Colorado Denver, Denver, CO, United States,; 8 Department of Psychology, Cornell University, Ithaca, NY, United States,; 9 Department of Psychological Sciences, Rice University, Houston, TX, United States,; 10 Department of Psychology, Stanford University, Stanford, CA, United States,; 11 Department of Psychology, McGill University, Montreal, QC, Canada,; 12 Department of Psychology, University of Southern California, Los Angeles, CA, United States,; 13 Counseling Psychology, University of Southern Mississippi, Hattiesburg, MS, United States

**Keywords:** community, emerging scholars, mental health, theory of change, well-being

## Abstract

Research increasingly points to well-being as a public priority, integral to the health of individuals and communities. In response to pressing societal challenges, many researchers seek to produce scientific knowledge that advances understanding and cultivation of well-being. Academic culture rightly uplifts the products of well-being research—the knowledge that improves people’s lives. However, relatively less attention is paid to fostering well-being in the process of conducting such research. Indeed, recent research indicating high rates of anxiety and depression (20%–50%) amongst graduate students and postdocs, suggest a troubling paradox: that the prioritization of well-being research outcomes may come at the expense of researchers’ own wellbeing. Early-career scholars, who assume substantial demands within the scientific enterprise, are especially impacted by this tradeoff. Yet, they are uniquely positioned to re-envision a praxis for research that embeds well-being in both its products and processes. Developed by an interdisciplinary coalition of early-career scholars, this perspective paper identifies key barriers to fostering well-being in research, and in turn, proposes four guiding principles and corresponding practices: 1) cultivate belonging, 2) center communities, 3) question cultural systems, and 4) embody cultural change. In turn, these principles aim to advance the societal impact of well-being research through community-based, participatory approaches that promote solidarity between researchers and communities historically excluded from the research enterprise.

## Introduction

There is a dynamic tension between the desire to promote well-being and the process of conducting well-being research within conventional academic ecosystems. Systemic obstacles to fostering well-being are, broadly speaking, endemic to academic institutions, with ramifications for researchers and communities alike (Hurd and Singh, 2021). These obstacles are infrastructural, cultural, and economic, and erode scholars’ experiences of equity, social connection, and financial stability, along with their capacities for authenticity, purpose, and self-care (Rahal et al., 2023; Smith and Ulus, 2020; Wilcox et al., 2021). Indeed, research suggests that graduate students are six times as likely to suffer from anxiety and depression relative to the general public (Evans et al., 2018), and graduate program attrition rates may be as high as 30%−50% depending on the field of study (Council of Graduate Schools, 2008). Given the inextricable linkage between researcher, community, and institutional well-being, the personal and political process of *how we conduct science* would benefit from a set of principles and practices that enable the conduct of a well-being research that truly reflects its subject matter.

This position paper proposes a set of four principles to promote cultures of well-being across higher education institutions and advance well-being as a research praxis. The guiding principles detailed herein emerged from a week-long workshop on emotional well-being held in summer 2023, which catalyzed the development of an *emerging scholars* consortium, committed to advancing a scientific system capable of promoting flourishing among researchers and communities impacted by the research. While previous work has advocated on behalf of emerging scholars (e.g., Kent et al., 2022)—a term colloquially used to describe early career researchers and educators, often at the graduate or postdoctoral level—such scholarship rarely prioritizes the viewpoints of emerging scholars themselves. This gap is noteworthy given that emerging scholars, although essential to academic productivity, are typically excluded from decision-making spaces (e.g., grant committees, faculty meetings, invited symposia). As funding prospects for emerging scholars continue to dwindle, with less than 2% of NIH-funding allocated to researchers aged 35 and under (Daniels, 2015), emerging scholars are afforded less and less power to shape the future of science. Given that graduate students and postdocs are centrally involved in processes of participant engagement and data collection, emerging scholars represent a unique class of stakeholders, with the perspective, motivation, and vision to re-imagine academic ecosystems.

Drawing upon the positionality of emerging scholars and an interdisciplinary body of research, we make the argument for adopting a set of principles for conducting well-being research. These principles are anchored in a shared *axiology*, or a *value orientation towards the practice of producing knowledge* (Guba and Lincoln, 2005). Such an approach to well-being science does not presuppose philosophical assumptions about the nature of well-being, a topic which has been extensively interrogated elsewhere (Park et al., 2023). Instead, we propose an orientation for conducting research grounded in four principles: *1) Cultivate Belonging*, 2) *Center Communities, 3) Question Cultural Systems, and 4) Embody Cultural Change*. We also offer practices to enact these principles, arguing that the embodiment of well-being should represent more than a rhetorical device (refer to Table 1). Finally, we advocate for the position that research is both personal and political, with care as a political stance that enables long-sought breakthroughs in well-being research.

### Positionality statement

As contributors to this piece, we carry multiple perspectives, identities, and experiences that have informed our collective epistemological position. It is insufficient and possibly essentialist to articulate how the many intersectionalities represented here have informed our axiology. A number of us write from lived experiences of marginalization as Persons of Color, as immigrants and international students, as those from lower socioeconomic backgrounds, or as those identifying as queer or neurodivergent. Likewise, many of us navigate from locations of privilege, either as descendents of European settlers or as those benefiting from the affordances of wealth, education, or heteronormativity. All authors were affiliated with U.S. or Canadian institutions at the time of writing, a positionality which admittedly limits our cultural perspective, particularly in regards to the experiences of emerging scholars in the Global South. A few of us write as community-engaged researchers who strive to elevate the perspectives of underrepresented communities, whose voices are all too often ignored in psychology research. The majority of contributors are trained in the philosophies and methods of Western Psychology, a discipline which has historically privileged post-positivist traditions (Rogers et al., 2024). Within the following sections, we seek to challenge assumptions of post-positivist traditions—particularly the assumption that scientific practice can be objective or value-free—and elevate alternative epistemologies of equal legitimacy (Held, 2023). We operate from a range of vocations, spanning research, clinical practice, and education. These career pathways undoubtedly shape our conceptualization of “academic institutions”, as well as our critiques and proposed practices for reimagining such systems. Our implementation of a team science approach and collective authorship is detailed in Supplementary Figure S1.

We recognize that we and our audience navigate a flawed, seemingly immutable system, reinforced by decades of cultural and economic forces and—more acutely—the prevailing powers of political administrations. Nevertheless, we strive to advance a more equitable and just future, as have generations of scholars before us from liberatory, indigenous, and Black Feminist traditions (Battiste, 2011; Collins, 1986; Martín-Baró, 1996). We aim to build on the inspiring work of these scholars, whose writings are prominently featured herein. The framework we propose is necessarily incomplete, aspirational, and dynamic. While we highlight multiple intersectionalities in our illustrative examples, our documentation of marginalized experiences in academia is far from comprehensive, and we uphold that all groups deserve elevation and access to culturally appropriate resources. This paper is intended to initiate dialogue that is situated in distinctly nuanced, continually evolving environments.

### An open invitation

We invite readers, both within and outside of higher education institutions, to engage with these ideas. While we anticipate that psychologists and education researchers will find this framework relevant to their work, the critiques and corresponding principles described here may feel most authentic to emerging scholars, regardless of their particular academic discipline. We also wish to invite into dialogue all readers who do not identify with being “academic”, especially those who have felt overlooked, excluded, or misunderstood by the educational system. Community members situated outside of academia have made invaluable contributions to research, often without compensation or recognition as co-creators of knowledge, by integrating cultural knowledge systems often neglected by mainstream research pedagogy. Herein, we assert that the advancement of well-being science critically depends on acknowledging the expertise of community members. Finally, we invite well-established scholars to join us in engaging with these ideas, some of which may be perceived as foreign or controversial. While there is always a personal and professional cost to resisting systems of oppression, cultural reform is possible through solidarity, and faculty afforded the protections of tenure may be uniquely positioned to challenge institutional norms and promote systemic change.

### A principles-based approach

**FIGURE 1:**
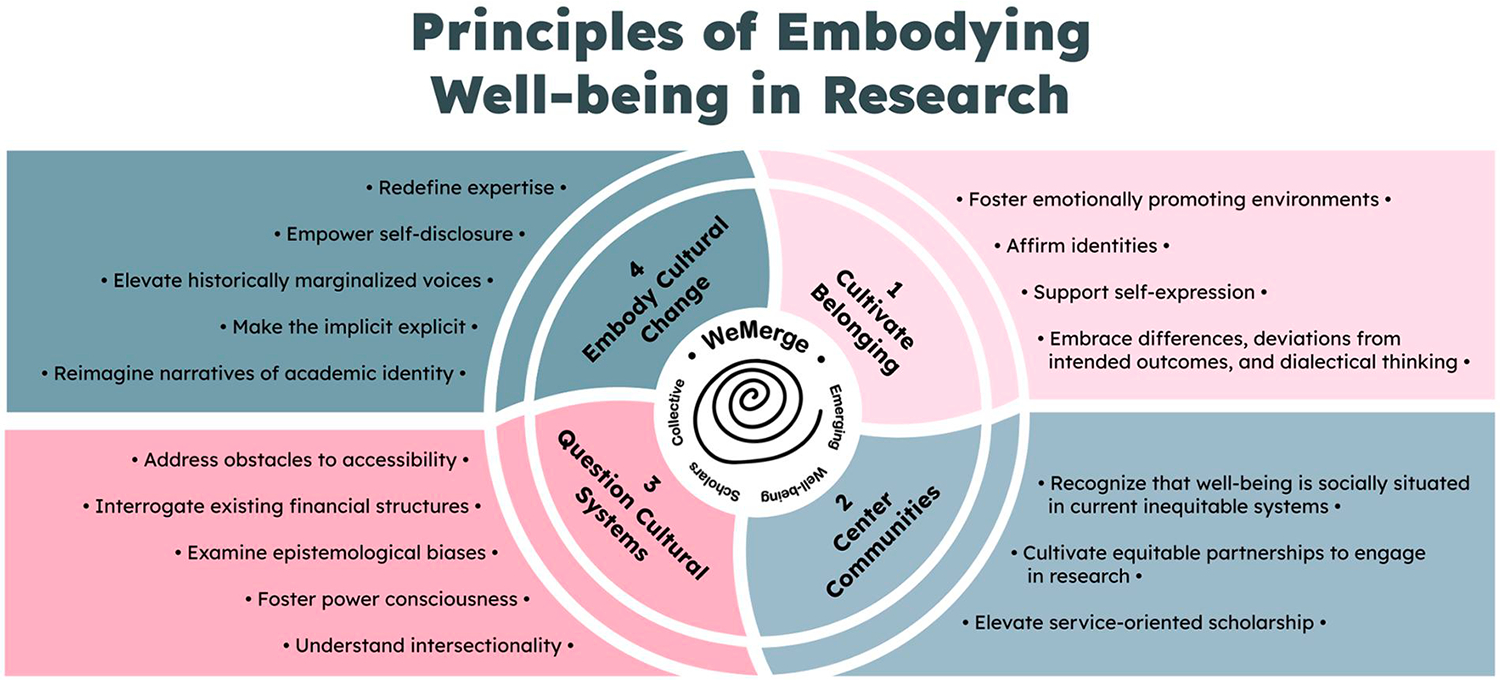
Organizational schema for the proposed framework for conducting principle-guided well-being research.

With this context in place, we present four guiding principles for advancing well-being research. We first focus on two principles grounded in relationality (Principle 1: Cultivate Belonging and Principle 2: Center Communities). With this foundation in place, we argue that it is only *through community* that we build the capacity for cultural change, which is the joint focus of principles three (Principle 3: Question Cultural Systems) and four (Principle 4: Embody Cultural Change). Figure 1 provides an overview of the four principles elaborated in the sections below.

## Principle 1: cultivate belonging

A sense of belonging entails a deep sense of connection and includes feelings of being accepted, included, and valued (Hagerty et al., 1992). The benefits of belonging, as well as the deleterious consequences of social isolation on emotional and physical health, are well-documented (Baumeister and Leary, 1995; Park et al., 2023). Belonging is critical to achieving equitable representation of scholars, given that a strong sense of belonging is thought to bolster fulfillment, achievement, motivation, and retention (Kahu and Nelson, 2018), while poor social connection has been associated with greater feelings of inadequacy, impostor syndrome, and anxiety that in turn threaten the retention of women and People of Color (Liu et al., 2019). In order to advance a scientific process that is sustainable, innovative, and ethically rigorous, it is crucial to first cultivate an environment that nurtures a sense of belonging.

### Foster emotionally promoting environments

We suggest that in order to cultivate a sense of belonging in young scholars, senior researchers and academic institutions must foster *emotionally promoting environments* (Lozada et al., 2022), or cultural spaces in which emotional expressions are validated as appropriate and meaningful. These environments allow researchers to fully experience and articulate a range of positive and negative emotions in a professional, academic context. However, such emotional expression is contingent on psychological safety, which may be established by principal investigators and faculty members empowered through their relative social capital. By definition, a psychologically safe space cultivates feelings of acceptance, where people are free to engage authentically, contribute ideas, and take action with the trust that all opinions and perspectives will be respected and valued (Edmondson and Lei, 2014). This freedom of expression can mitigate feelings of isolation for People of Color and neurodivergent scholars, for example, who may feel alone in navigating their personal identities.

Effective mentorship plays a key role in fostering a sense of belonging among emerging scholars. In order to support emerging scholars to work independently from a space of trust and psychological safety, this complex relationship should recognize the importance of effective communication informed by equity and inclusion (Pfund et al., 2013). For example, mentors can safely validate the emotions of trainees by communicating that each setback or “failure” is important for learning and growth. Notably, mentorship that effectively meets the needs of minoritized students requires significant time commitment, which inherently compromises activities required for promotion and tenure. Thus, to systematically shift the culture of mentorship in academia, excellent mentorship must be recognized and rewarded in the tenure and promotion process. Universities can support this process by evaluating mentorship quality as equally meritorious to academic productivity. On the other hand, consistently inadequate mentorship should not be tolerated without remediation, even from highly productive, innovative individuals with top credentials.

Mentors can also encourage emotionally promoting environments by *modeling and embracing vulnerability* (Bettez, 2017). Embracing vulnerability involves sharing personal stories, acknowledging one’s own limitations, and demonstrating openness to feedback. By modeling vulnerability, senior researchers create a culture of trust and courage, which empowers emerging scholars to engage authentically in their work. In turn, such authenticity can promote creativity, innovation, and collaboration. Exchanging personal stories that acknowledge one’s shortcomings can also counter traditional academic narratives of hyper-competition and build space to imagine a healthier, more inclusive academic culture. However, such vulnerability should be practiced with sensitivity and without creating pressure for self-disclosure, given that self-disclosure may not always be safe or desired by community members with less power or traditionally underrepresented identities.

### Affirm identities

Affirming identities refers to the active support and recognition of individuals’ diverse backgrounds and experiences, particularly in academic settings, and is critical for cultivating feelings of affiliation and acceptance within a given group (Teng et al., 2020). Affirming the identities of underrepresented scholars requires fighting stereotypes and biases in academic spaces and investing in programs and resources that promote mentorship, networking, and professional development opportunities for all (Casad et al., 2021). Unfortunately, many academic spaces currently do not affirm diverse identities and researchers from marginalized communities continue to be underrepresented and face obstacles in pursuing and building research careers (see Chavez and Weisinger, 2008 for an overview and recommendations). For instance, women in the United States leave faculty jobs at consistently higher rates compared to men (Spoon et al., 2023), with such women reporting little sense of belonging within their departments or experiences of harassment. Similarly, in the European Union women comprise less than 27% of the highest tier academic positions (European Commission, 2021).

When we affirm identities, we communicate to scholars from underrepresented communities that they offer valuable scientific insight and are uniquely positioned to advance the research priorities of their communities (Juang, 2024). Embracing the diversity of perspectives within research not only fosters inclusivity and a stronger sense of belonging among researchers but also drives innovation by valuing different methods, and insights, ultimately enriching the academic discourse and promoting a more comprehensive understanding of the world in academia. By recognizing these scholars’ strengths—in their approaches to data collection, interpretation, and theoretical development—we can affirm their identities (as women scholars, scholars of color, etc.) and celebrate diverse perspectives as essential to scientific growth. Given that aspiring researchers are more likely to envision themselves in research careers when they see successful researchers with similar backgrounds and experiences (Estrada et al., 2018), departments can further affirm diverse identities by providing resources for mentorship and network building with scholars of representative backgrounds.

### Celebrate differences

*Celebrating differences* in background and positionality, including differences in race, ethnicity, life experience, and neurodiversity, requires actively valuing the unique insights that different perspectives bring to academic discourse. Diversity has been empirically linked to scientific innovation, with research showing that scholars from underrepresented groups contribute to novel research at higher rates (Hofstra et al., 2020). Thus, we argue that diverse perspectives should be championed and afforded culturally-appropriate support to redress the “diversity-innovation paradox in science”, or the systematic discounting of novel research conducted specifically by minority-group scholars (Hofstra et al., 2020).

We suggest that *honoring disagreements* can increase appreciation for diverse perspectives by viewing differing opinions as essential for a deeper, nuanced understanding. When approached with respect and openness, recognizing and honoring disagreements can catalyze innovation by encouraging critical thinking and challenge researchers to consider alternative methodologies (Turner et al., 2015). When differing epistemic values and methodologies are viewed as valid sources of knowledge production, and approached with commitment to ongoing learning, tensions between intellectual perspectives can be embraced as valuable sources of scientific progress (Turner et al., 2015). While challenging, honoring disagreements may be supported through formal communication practices such as *collective decision-making*, a process by which group members–irrespective of their position of power—come to a consensus after interacting, discussing, and reaching an agreement on how to move forward. With practice and experience, this process can reduce group conflict, empower group members, and promote cooperation (Linehan and Wilks, 2015).

Despite the creative advantages associated with diverse research teams (Wang et al., 2019), underrepresented minorities are at risk of leaving academia, often due to feelings of inadequacy in academic success or lack of belonging (Kricorian et al., 2020). Building a sense of belonging is foundational for cultivating environments that support collaboration and diversity. However, to sustain well-being and equity in research, it is equally important to extend this belonging beyond academic settings. By doing so, we can create relational networks that not only support individual scholars but also establish equitable partnerships with the communities impacted by research efforts.

## Principle 2: center communities

Drawing from principles of community psychology, we contend that science is necessarily *relational*, as the generation of knowledge is collaborative and contextual (Hall et al., 2018), and is heavily informed by social and cultural norms. In alignment with stakeholder- and community-engaged approaches (e.g., Ahmed and Palermo, 2010), we propose that effective well-being research requires building relationships between scholars and the people who will be impacted by, implement, and/or disseminate the research. However, envisioning the equitable redistribution of resources and expertise—thereby foregrounding the relational nature of science—remains a challenge for many established researchers. The majority of psychology research still uses non-participatory methodologies (i.e., research paradigms that lack incorporation of stakeholder perspectives) and organizational systems that concentrate expertise and power around high-ranking or tenured scholars (Rodriguez Espinosa and Verney, 2021). Here, we illustrate what it means to *center communities*.

### Recognize that well-being is socially situated in current inequitable systems

Disparities in well-being outcomes mirror those of health outcomes (Diener et al., 2017), both of which are symptoms of broader systemic inequities (Plamondon et al., 2023). A wealth of epidemiological research shows that structural inequalities place marginalized populations at greater health risk (Krieger, 2020), and that the well-being of such groups is disproportionately threatened by prolonged stress exposure from social, financial, and environmental threats (Forde et al., 2019). When well-being *for all* is the goal, it prompts us to return to the very beginning of the research process: how are researchers developing theories and asking questions? From the start, research must consider inequities that shape well-being and underlie pressing social problems. One advantage of centering community perspectives at the earliest stages of research is that it allows participating communities to define well-being in terms of the resources and opportunities with their unique ecologies (Arcidiacono and Di Martino, 2016), and by foregrounding these contexts, enables research to engage more directly with policy.

Public psychology refers to applying psychology to solve social problems and improve people’s lives (Eaton et al., 2021). It highlights the discipline’s potential role as a driver of positive social change. This lens also draws attention to ways in which psychologists have colluded in structural oppression and avoided structural power dynamics. Centering social problems in research foregrounds ethics underlying social transformation and justice (Eaton et al., 2021). While such “applied” research is often separated from pursuing “basic” science focused on fundamental understanding, the paradigm of use-inspired basic research transcends this dichotomy (e.g., Wilson-Mendenhall and Holmes, 2023). This framework illustrates that basic and applied research goals can be pursued simultaneously. When applied use is a goal of basic science, the research begins with a social issue, as a starting point, and thus the aforementioned ethics involved in studying social problems (Wilson-Mendenhall and Holmes 2023). As we turn to next, ethical frameworks of social justice and transformation require researchers to go beyond social issues as inspiration and turn toward deep involvement with communities directly impacted by the research.

### Cultivate equitable partnerships to engage in research

As scholars, it is important to acknowledge how researchers have—and in some cases continue to— conduct science *about* marginalized populations *without* the permission and engagement of impacted communities. In 2021, the American Psychological Association (APA) issued an apology for the field’s contributions to promoting ideologies of racial supremacy, minimizing the contributions and concerns of psychologists from communities of color, and insufficiently reporting data from communities of color (Akbar et al., 2024). Nevertheless, many high impact journals continue to dismiss epistemologies and methodologies from non-Western traditions, and practices of tokenism and extractionism are perpetuated in study designs, data collections, and interpretations (Shahram, 2023). We argue that research that is likely to benefit *well-being for all* is research that prioritizes stakeholder engagement. Nevertheless, research in this domain is scarce and underscores a broader trend of low stakeholder engagement in psychological science (Rodriguez Espinosa and Verney, 2021), possibly due to intensive time and energy commitments inherent to building community-research partnerships.

We argue that deep community involvement—not just academic expertise—should guide the research process to ensure real-world impact. Specifically, we advocate for equitably partnering with marginalized communities throughout all stages of the research process, from grant development to dissemination and implementation, and the institution of community advisory boards (CAB) as formalized research-community partnerships (for a synthesis of best CAB practices, refer to Newman et al., 2011). This practice of community-engaged research offers an evidence-based approach to aligning research with public values, incorporating diverse perspectives, and communicating findings in ways that are accessible and beneficial to communities (Eaton et al., 2021). In fact, such stakeholder engagement has been effectively used to advance the well-being of emerging scholars to develop the Researcher Toolkit (Homer et al., 2021), a peer-support mental health program co-created by U.K. postgraduate research students. As an initial step towards community engagement, researchers can embody cultural humility by critically examining Euro-American psychological narratives, derived from globally non-representative samples. Cultural humility lays the groundwork for the equitable co-creation of knowledge in ways that leverage expertise from all partners. In turn, these approaches can inform policy and accessible health interventions to empower affected communities (Collins et al., 2018).

### Elevate service-oriented scholarship

Robust community engagement continues long after data collection is completed, and extends to the domains of knowledge translation, implementation, and policy advocacy (e.g., Lawrence et al., 2019). Communicating research in ways that are accessible may improve the “research to practice gap”, wherein many effective psychosocial interventions are not adopted at the population level (Kazdin, 2017), often because dissemination to community spaces is limited or because such research fails to resonate with the goals of community stakeholders. Although science communication guidelines exist to facilitate knowledge accessibility (e.g., National Institutes of Health, 2018), we may enhance research impact by investing in scholar-practitioners who directly interface with target communities. Furthermore, community-research partnerships can support inclusive knowledge translation through policy briefings, media, local events, etc. Notably, the reach and impact of such research depends on *policy*. Thus, researcher collaboration with policymakers can actualize the immediate benefits of well-being research for vulnerable communities.

As a final note, we argue that community-engaged research depends upon the support and solidarity of academic institutions. Building a culture of community engagement is challenging, especially when navigating an increasingly tumultuous political landscape. Meanwhile, institutions and their researchers must contend with the pressures of research productivity, which threaten to overshadow time-intensive service-oriented scholarship. Some researchers have called for institutions to formally incentivize service-oriented scholarship, thereby publicly signaling their support (Jessani et al., 2020). However, other scholars have expressed concerns about incentivizing advocacy, fearing that visible advocacy may invite reputational damage or compromise the personal safety of scholars. Acknowledging these legitimate apprehensions, we nonetheless endorse institutional mechanisms aimed to advance service-oriented scholarship. Institutions can signal support for this work through financial incentives, including the provision of discretionary funding, salary relief, and internal grants allocated towards relationship-building activities. Institutions can also elevate the status of such scholarship by extending awards for excellence in service, providing workshops targeting service-oriented skill sets, and increasing the merit of service as a tenet for promotion. Beyond the institution level, networks of citizen science practitioners, such as those organized across Europe, Australia, and the U.S., can build capacities for initiatives that deepen public engagement and catalyze policy development (Hecker et al., 2018).

## Principle 3: question cultural systems

While principles 1 and 2 offer a vision of well-being research that is inherently relational, principles 3 and 4 build upon these concepts to identify leverage points for cultural change. We argue that research is necessarily embedded in relational and institutional systems, influencing practices of conferring and restricting power. Scientists are trained to think critically about many facets of their research. Here, we offer an opportunity to train this critical lens on the systems that—while difficult to perceive—undoubtedly influence our livelihoods and society more broadly. Briefly examining the cultural and economic forces that shape academic systems, we invite readers to “dig deep” and reflect upon their own experiences within these systems.

### Grapple with the economics of academics

The modern university system predominantly operates as a global economic enterprise in which knowledge is a measurable commodity and knowledge workers are a managed resource (Steck, 2003). As business entities, universities aim to maximize research revenue and minimize employee costs. Diminishing state funds have pressured universities to adopt corporate strategies, warping the academic labor market to reduce the number of tenure-track professorships and increasing the number of low-cost knowledge workers in the form of graduate students and postdoctoral researchers (Feldon et al., 2023). Consequently, far fewer graduate students and postdocs will ascend to tenured faculty positions. This state of job scarcity makes students and postdocs susceptible to labor exploitation; tenure-track principal investigators critically rely on the inexpensive labor of these emerging scholars to continue receiving grant-funding, and emerging scholars may endure exploitative practices to enhance their own prospects in an increasingly precarious labor market (Feldon et al., 2023). Meanwhile, increasing demands for peer-reviewed publications, which universities’ depend upon to attract public funding, has contributed to a “peer-review crisis” and “reviewer fatigue” (Breuning et al., 2015; Horta and Jung, 2024) characterized by overburdened researchers with little time or incentive to engage in the free, anonymous labor of peer-review. Consequently, emerging scholars feel strong pressure to publish (or perish), yet contend with long delays post-submission, high open access fees, and frequent requests to volunteer their own time for peer review (Flaherty, 2022).

The adage that low-earning graduate students are simply “paying their dues” belies the genuine human costs of this financial arrangement. Graduate students and early career researchers must grapple with historically high student loan debt. In the U.S., psychology graduate students are estimated to owe $110,000 (median estimate) in cumulative student loans (Doran et al., 2016). Managing such debt (among other financial stressors) with low-wage stipends (approximating $20,000–30,000 annually) has made pursuing a graduate education increasingly untenable, especially for students with marginalized identities who borrow at substantially higher rates and are disproportionately impacted by “hidden” professional costs of academia (e.g., self-funded professional attire, conference fees and travel, etc.) (Wilcox et al., 2021). With an expected median income of $60,000 upon graduation, prospective students incur significant economic risk, often delaying other valued life milestones (e.g., having children, saving for a home or retirement) (Doran et al., 2016). Such financial strain will effectively “price out” many individuals who—while highly capable and intrinsically motivated—cannot bear the very real financial, physical, and emotional burdens inherent to this arrangement. Moreover, these financial barriers arise long before graduate school; while research experience is requisite for a successful graduate school applicant, the vast majority of research assistantships available to undergraduates and post-baccalaureate individuals are unpaid (Coté, 2023). Consequently, many research assistantships are simply inaccessible to low-income “working students”, who face inordinate time constraints to support themselves financially.

Postdoctoral and early career researchers without fixed employment face similar yet unique financial challenges due to the precarity of their employment. The U.S. has become increasingly reliant on the temporary employment of postdocs; from 1998 to 2007, the number of postdocs increased by 21% (Hoffer et al., 2008) while the number of tenured faculty positions has remained relatively stable. By 2021, it was estimated that 53% of all U.S. postdoc positions were filled by international employees. With postdocs earning a median annual salary of just $49,000 (Gewin, 2023), this low-cost, migratory workforce is vulnerable to exploitation, and many postdocs linger in these “temporary” positions for many years, often with minimal benefits and insufficient childcare. The precarity of academic labor is not restricted to the U.S., but rather is observed as part of a larger globalized phenomenon of academic capitalism (Feldman and Sandoval, 2018; McCarthy et al., 2017; O’Connor et al., 2024; Shin et al., 2018; Welch and Li; 2021); although the number of postdoc positions and representation of women appear to be relatively diminished in the Global South (Khadakkar and van Nouhuys, 2024).

Dominant narratives in academia frame personal burden—whether financial, physical, or emotional—as a willing choice of “good academics” who demonstrate their virtue through self-sacrifice. Regardless of whether or not this narrative is consciously understood, it is certainly weaponized to minimize the unfair labor conditions of emerging scholars. Though academic work is undeniably privileged work, offering for many a sense of meaning and intellectual satisfaction that is uncommon in today’s industrial workforce, this privilege comes at tremendous cost. Universities have become virtually dependent on unpaid labor, despite the fact that this sacrifice is virtually impossible to maintain, particularly for parents, caretakers, and those with financial, medical, or emotional hardship. Without changing the financial systems of the modern university, access to positions of power (i.e., tenured faculty) will remain inaccessible to all but those with greatest privilege.

### Examine epistemological biases

Broadly defined, “science” refers to the systematic method of producing knowledge; however, in mainstream research, this term is typically used in reference to modern Western Science, a practice that emerged within a specific cultural and political context (e.g., Enlightenment era Europe), and from such contexts, became defined by a specific set of philosophical values (Held, 2023), namely that truths are objective, universal, and observable through falsifiable hypothesis testing. In modern Euro-Western psychology, these assumptions are central to the epistemological framework referred to as postpositivism, a tradition which has withstood decades of critique and reinterpretation (Holtz and Odağ, 2020). Notably, numerous psychological traditions—including feminist, liberatory, and decolonized psychology traditions—assert that postpositivism allows for methodological pluralism (Grzanka and Cole, 2021). These traditions stand apart from mainstream Euro-Western traditions by challenging assumptions that scientific methods are inherently ideologically neutral. Indeed, history demonstrates how science, under the influence of dominant sociopolitical forces, has been used to justify the legitimacy of hegemonic powers (e.g., white supremacy, heteronormativity) (Lala and Feldman, 2024). Nevertheless, the portrayal of science as an objective, purely rational practice, prevails in mainstream academics (Grzanka and Cole, 2021; Rogers et al., 2024). In turn, mainstream psychology has adopted the narrative that “good science” (Rogers et al., 2024) excludes methods that do not make similar assumptions about falsifiability, generalizability, and universality. Following this, other epistemologies and methodologies—those that adopt value-based ethics and operate under assumptions of subjectivity and contextual sensitivity—have become marginalized from the discipline.

Inclusion cannot be accomplished simply by improving representation of marginalized communities. Inclusion means acceptance of methods designed to humanize these communities and elevate their wisdom to the status of expertise. Indeed, prior research suggests that the integration of qualitative, participatory methods can increase engagement of marginalized populations, empowering such groups through freedom of expression (Pincock and Jones, 2020) and foregrounding cultural values (Finlay-Smiths et al., 2024). When leading scientific platforms (e.g., journals, departments, and institutions) routinely exclude qualitative research or imply that such methodologies are “lacking in scientific rigor”, a political claim is being made about whose knowledge and ways of knowing count as legitimate (Grzanka and Cole, 2021; Settles et al., 2021). This practice silences perspectives (e.g., intersectional, queer, feminist) that challenge erroneous (and sometimes oppressive) assumptions of mainstream science, which in turn stifles scientific growth. While studies using *a priori* hypotheses, experimental control, and empirical approaches are of undeniable value to knowledge advancement, we argue that a science of well-being must also recognize the value of methods that honor positionality, relationality, and context, noting that such methods have advanced psychological knowledge by illuminating false assumptions about underrepresented experiences (Lewis, 2021). In turn, we suggest that by practicing epistemic humility, scientists can incorporate elements of other established epistemologies (e.g., Feminist, Constructionist, Indigenous) in circumstances necessitating context sensitivity (over internal validity) or when a phenomenon of interest is highly complex, assumptions are poorly understood, or evidence is insufficient for hypothesis testing.

The advantages of methodological pluralism are illustrated in the following case examples situated in Aotearoa New Zealand, a country in which approaches to research have been historically shaped by Te Tiriti o Waitangi, a treaty negotiating partnership between the Māori and British Crown and extending to all Crown Research Institutes (Finlay-Smiths et al., 2024). Following initiatives to uphold the Te Tiriti in ways that support research by and for Māori, the He Wai Māpuna programme at the Institute for Environmental Science and Research (ESR) was developed as a community well-being program that conceptualizes water as a well-being priority in alignment with Māori ways of knowing (Finlay-Smiths et al., 2024). In order to successfully integrate Western and Māori environmental methods, researchers needed to transform their epistemological assumptions, recognizing the nature of knowledge as communal holistic (e.g., reflecting the interdependence of environments, ancestors, and spiritual sources) (Edwards, 2026). Applying a similar approach, the *Te Ara Tika* strategy of agricultural research foregrounds Māori principles as well as historical, political, and social context to innovate agricultural research in ways that address intergenerational inequalities (Finlay-Smiths et al., 2024). He Wai Māpuna and *Te Ara Tika* are only two of the many examples of methodological pluralism practiced globally, and while a review of such projects is beyond the scope of this paper, these research initiatives illustrate the social impact potential of epistemic humility. Finally, it warrants emphasizing that non-postpositivist epistemic traditions possess distinct criteria for evaluating validity and rigor (e.g., member checking, triangulation, field exposure), and that learning these methodologies often requires lengthy mentored training or the collaboration of practitioners.

### Foster power consciousness

Within academic spaces, power is commonly organized in a vertical hierarchy, meaning that expertise, resources, and authority are concentrated at “top” levels of the hierarchy occupied by tenured faculty or similarly high-powered individuals. While vertical hierarchies effectively maintain role clarity and support short-term productivity, this distribution of power may disadvantage emerging scholars and eventually hinder scientific progress (Friedensen et al., 2024; Xu et al., 2022). Because vertical hierarchies encourage individualism, this arrangement can minimize the shared contributions of junior scholars and risks inhibiting the professional autonomy of trainees. Consequently, emerging scholars may struggle to maintain intrinsic motivation, a quality many consider to be the lifeblood of intellectual innovation. In worst-case scenarios, emerging scholars may be vulnerable to microaggressions and even overt acts of discrimination (Friedensen et al., 2024). In order to support the well-being of emerging scholars, we advocate for the use of “power-conscious frameworks”, which address the structures and expressions of power in higher education (Friedensen et al., 2024). Evidence suggests that disrupting hierarchical relationships may measurably improve research impact. In an analysis of over seven million co-authored papers published between 1980 and 2020, researchers found that research teams with relatively equal power distribution (i.e., “flat” power structure) and greater leadership sharing produced higher impact research relative to hierarchical teams (Xu et al., 2022, 2024).

The importance of power sharing likewise applies to scientific partnerships with historically disempowered communities. If left unexamined, the hierarchies of power observed in academic spaces are reproduced in community-researcher relationships. Researchers, who hold access to institutional resources and are themselves symbols of privilege and status, must deliberately redistribute their power in order to build trust with communities and participate in the co-creation of knowledge (Muhammad et al., 2015). Power discrepancies may also be recapitulated through covert practices (e.g., the overuse of academic language), or micro-level behaviors that serve to enable macro-level inequalities (Friedensen et al., 2024). In sum, power dynamics impact all levels of research praxis, and we must recognize the influence of such power structures to prevent the misuse of academic power within and beyond our institutions.

### Recognize intersectionality

Although the term intersectionality is often used as a synonym for multiple identities, intersectionality more accurately describes how access to power emerges from the interaction of one’s environment and identity (Warner, 2008). Learning to confront power imbalance begins with understanding relationships and recognizing how status is continually conferred or performed through relations embedded within organizational cultures. To achieve academic success, trainees must navigate numerous complex relationships with mentors, mentees, peers, collaborators, funders, community partners, etc., a process made all the more challenging when identities are not shared or appreciated. Racially and ethnically minoritized trainees often contend with stereotype threat, and mentors with low cultural competence may err in the assessment, training, and ultimately the well-being of these trainees (Carrero Pinedo et al., 2022). Power is also conferred or reified at the organizational level. Although many academic departments openly promote diversity, equity, inclusion, and accessibility, they may only implement solutions that conform to established power structures (Ahmed, 2012). Thus, initiatives aimed at merely increasing representation maintain the illusion of equity promotion, but fall short of addressing cultural norms or structural factors that uphold inequities.

Finally, intersectionality illuminates how an individual’s positionality to systems of power and oppression shape their ability and motivation to uphold or upend cultural practices. For example, faculty of color, queer faculty, women faculty, and faculty from the working class experience a higher burden of responsibility to address diversity-related departmental and university issues (Joseph and Hirshfield, 2011). Accordingly, these faculty spend a disproportionate amount of time on “invisible” service while being expected to fulfill the same teaching and research requirements as their majority-culture peers. This form of “identity taxation” (Joseph and Hirshfield, 2011) has been shown to negatively impact tenure and promotion rates. Notably, the burden of diversity-related work has drastically escalated in the last several years due to increases in “anti-woke” political rhetoric across Europe and the U.S (Samaras, 2025)., as well as U.S. state- and federal-level “DEI bans” which seek to comprehensively eliminate policies, practices, research, and rhetoric associated with diversity, equity, and inclusion. In turn, scholars already burdened by the responsibilities of diversity-related labor must also bear greater personal and professional risk in the form of negative teaching evaluations, manuscript rejections, grant cancellations, denial of tenure or promotion, racial battle fatigue, and even open harassment (Dhanani et al., 2024).

## Principle 4: embody cultural change

While people are shaped by their cultures, they are also active participants in upholding or resisting cultural norms. The previous section raises awareness of the systems that define scientific praxis, and likewise, invites self-reflection on how we as individuals influence these systems with respect to our unique positionalities (Hamedani et al., 2024). This final section petitions readers to transform these reflections into actions by illuminating leverage points within our own relationships, communities, and scientific practices (refer to Table 1 for specific applications). In this vein, we underscore that principles adopted in earnest are inherently *embodied* or *enacted*.

### Make the implicit explicit

Every academic setting has its own set of social norms, expectations, and markers of professional identity and skill. Much of the training in academic settings involves development of social norms and customs, a process that is entrained through mentorship, expected in culture, but not ever stated or explained (Hopkins et al., 2024). Although many of these norms are treated as markers of competence and success, they are not made explicit but rather are transmitted subtly in what has been described as the “hidden curriculum” (Hopkins et al., 2024). This may be referred to as “soft skills” or “fit” which do not reflect well defined constructs, but are subjective words often used to describe how they are adhering to the hidden curriculum. In some cases, these implicit expectations can be positive, (e.g., an organizational culture of work-life balance, collegiality); however, such expectations can also sustain harm by reinforcing inequalities or unhealthy work-life balance.

Given the prominence of white supremacy culture in mainstream academic institutions, hidden curricula often promote white culture and center white experience (Carrero Pinedo et al., 2022). When a trainee’s foremost identity does not conform with implicit expectations, they may be viewed as “unprofessional”. In such respects, the hidden curriculum can insidiously perpetuate inequities as these biases go unacknowledged without opportunity for challenge or rectification. Consequently, failure to fully understand or internalize implicit expectations can obstruct academic progress, even when trainees demonstrate high academic capability. Notably, disadvantages stemming from the hidden curriculum are unequally distributed, with international students, first-generation students, and racial-ethnic minority students at heightened vulnerability (Andrade, 2006; Stephens et al., 2012).

### Empower self-disclosure

Earnest efforts to support inclusion require an equitable and safe process for self-disclosure. While much of the literature on self-disclosure pertains to the disclosure of disability or illness, self-disclosure also applies to those with other marginalized identities, either visible or invisible (e.g., race-ethnicity, nationality, sexual orientation, etc.) who may engage in identity disclosure to establish legitimacy and self-worth, particularly when their research implicates one or more of their identities. Self-disclosure is a highly nuanced and fluid process connected to how the person making the disclosure understands and negotiates their identity (Pearson and Boskovich, 2019). In the context of disability, identity disclosure means petitioning for the resources and services universities must provide while balancing the risk of stigma from peers, faculty, and administration (Cole and Cawthon, 2015; de Cesarei, 2015). Thus, disclosure is inherently a *relational* process that necessarily acknowledges identity and power in academic spaces. While self-disclosure may initiate *formal* processes of providing needed services to students and trainees, the well-being of these individuals likewise depends on *informal* social-emotional exchanges embedded in an organizational culture that can either validate or stigmatize identity disclosure (Pearson and Boskovich, 2019).

Whether or not students self-disclose depends upon how students believe their peers and supervisors will evaluate their identities. Students may withhold disclosure—instead attempting to “pass” or assimilate—due to fears of negative evaluation, shame, or discrimination, which may indirectly contribute to feelings of depression, loneliness, and invisibility (Lewis, 2021). However, disclosure may also be a potent tool for disrupting traditional power structures in academia, thereby opening the door to individuals who have been otherwise historically excluded and marginalized. For example, disclosure can foster dialogue around equity and collective responsibility while empowering students, mentees, or community members to view themselves as experts by virtue of their lived experience. By sharing thoughts and feelings around one’s identity, disclosure can also support self-compassion and self-esteem, which jointly mediate the relationship between disclosure and psychological resilience (Harvey and Boynton, 2021). Nevertheless, because self-disclosure requires “making the private public” (Pearson and Boskovich, 2019), without psychological safety, self-disclosure can be emotionally exhausting or exploitative. For this reason, different teaching pedagogies, such as those borrowed from social work (Segev and Hochman, 2023) and feminist pedagogies (Borshuk, 2017), have begun to integrate strategies to safely encourage self-disclosure. Such strategies include honoring personal experiences as a source of knowledge while stressing the importance of relational boundaries and confidentiality, and priming classroom communities with perspective-taking cognitive skills (Borshuk, 2017).

The principles of self-disclosure enumerated thus far may also apply to the conduct of research with populations disproportionately impacted by ongoing and historical trauma. For Persons of Color, traumas embedded in colonialism and systemic oppression may be reified in academic spaces, resulting in harms that often escape the attention of faculty and student peers (Voith et al., 2020). Thus, we advocate in favor of trauma-informed praxes, which prioritize safety, autonomy, trust, and empowerment (Voith et al., 2020). Fostering these qualities may enable researchers and community partners to acknowledge and transform the harms enacted upon historically marginalized communities by academic institutions.

### Elevate historically marginalized voices

Creating a more equitable psychological science requires elevating the voices of individuals from historically marginalized communities, especially across scholarly outlets. Unfortunately, ignoring, discrediting, or devaluing knowledge from these communities is endemic to psychological research, and formally perpetuated through practices of producing, reporting, and disseminating science in ways that uphold gatekeeping (Buchanan et al., 2021). In turn, such practices may contribute to discouragement and attrition of underrepresented scholars as well as the persistence of health and well-being disparities in marginalized populations. The systematic exclusion of racially and ethnically minoritized perspectives is empirically well-documented. Evidence demonstrates that manuscripts highlighting race are rarely published in top-tier psychological journals, and those that are accepted for publication are predominantly written by White authors (Roberts et al., 2020). Consequently, the works of minoritized scholars are more likely to appear in lower-ranking journals, impacting race-based disparities in citation scores. Furthermore, across all disciplines, scientists from non-western countries are relatively less likely to be cited (Gomez et al., 2022).

Counter to policies disparaging DEI measures, we argue that scholarship from historically marginalized voices is imperative to the construction of knowledge. Recentering underrepresented perspectives requires commitment to expand the scope of methodologies suited to strengths-based inquiry (e.g., person-centered quantitative methods, mixed-methods approaches, CBPR) (Buchanan et al., 2021), and necessitates buy-in from publishers, editorial boards, federal granting agencies, and reviewers. Despite these challenges, elevating historically marginalized voices in psychological research can challenge systems of oppression, with notable implications for clinical practice and social advocacy (French et al., 2020).

### Reimagine narratives of academic identity

As previously discussed, the hidden curriculum of academia often reinforces the implicit belief that one’s value is measured by external accomplishments, as narrowly defined by a particular set of neoliberal values (Hurd and Singh, 2021). This conceptualization of a “good researcher” is not of a person who contributes to and draws support from their communities and own lived experiences, but is instead often narrativized as the stereotypical “brilliant, lone scientist” — a person dedicated to their work and to productivity at the detriment of all else. This narrative becomes internalized, normalizing self-sacrifice and alienation as part of the culture of academia. It also undermines efforts to engage in interdisciplinary and fulfilling collaborations. We seek to reimagine narratives of what makes a “good researcher” to include qualities beyond individualist ambition and productivity.

Measuring research success solely as research output—through publication counts, citation metrics, and grant funding—overlooks broader contributions, qualities (e.g., determination, creativity), and collaborative relationships essential to addressing complex challenges and advancing innovation. More insidiously, this practice facilitates a culture of pressurized research performance characterized by competition, insecurity, and diminished self-worth (Smith and Ulus, 2020). These pernicious success narratives may be unwittingly reproduced in relationships with other scholars. At all career stages (and in all disciplines), scholars look to their advisors and advisees to inform their identities and evaluate their own academic merit. Thus, as scholars, we hold a collective responsibility to shape academic narratives in the service of well-being, and by extension, scientific progress.

Many of the most pressing scientific questions require multidisciplinary approaches and collective efforts. However, this work necessitates resisting mainstream narratives of academic success, and indeed, movements of resistance against “metric cultures” that prioritize economic productivity over social responsibility have emerged amongst dissatisfied UK scholars (Feldman and Sandoval, 2018). Science and the researchers who conduct science, do not exist in a vacuum. Thus, values of curiosity, collaboration, and collective responsibility must be encouraged at a cultural and institutional level. As cultural ideas are embodied in institutions, narratives offer a powerful medium for cultural change. By reclaiming our own narratives as emerging scholars, we may begin to reimagine a better way to practice research that enables well-being for all.

## Conclusion

By prioritizing equity, redistributing power, and embracing diverse perspectives, these principle-based guidelines are intended to transform well-being science into a field that not only generates impactful knowledge but also supports the flourishing of all stakeholders involved. We invite readers, from spaces within and beyond academic institutions, to engage with these ideas, reflect on their applications, and contribute to a collaborative vision for research to advance well-being for all. This is an aspirational, ongoing effort and will require collective commitment to reimagine the systems, cultures, and practices that shape the production of knowledge.

## Supplementary Material

Figure S1

The Supplementary Material for this article can be found online at: https://www.frontiersin.org/articles/10.3389/feduc.2026.1836519/full#supplementary-material

## Figures and Tables

**TABLE 1:** Main principles with corresponding practices and stakeholders.

Main principle	Practices (actions)	Applicable to principle (s)				Stakeholders				
1. Cultivate Belonging	Promote compassionate and supportive mentorship by incorporating mandatory mentoring training for advisors as part of the tenure and promotion process (APA Task Force on Race and Ethnicity Guidelines in Psychology, 2019).	1					A		D	
	Intentionally design and facilitate spaces that foster connections among scholars and promote psychological safety during professional meetings (Edmondson and Lei, 2014; Kricorian et al., 2020). Embrace transparency and humility by sharing personal stories, acknowledging one’s own limitations, and demonstrating openness to feedback (Bettez, 2017).	1		3	4	S	A	R	D	
	Support cultural competence and address personal biases through institution-supported training and structured discussion (Betancourt et al., 2003; Casad et al., 2021).	1		3	4	S	A	R	D	
	“Bring your whole self to research” (and enable others to do so on their own terms) by foregrounding emotions, identities, and lived experiences within the scope of professional spaces and activities (Hordge-Freeman, 2018).	1		3		S	A	R		
	Celebrate differences and empower historically underrepresented scholars by actively recognizing their contributions and providing opportunities for their advancement (De Dreu and Weingart, 2003; Nemeth, 1986).	1	2		4	S	A	R	D	C
2. Center Communities	Establish equitable partnerships with community stakeholders by exercising cultural humility, recognizing expertise of community partners, and increasing community operational capacities (Williamson et al., 2020). Co-develop research agendas with community partners through regular feedback sessions.		2	3	4			R		C
	Establish ‘deep collaboration’ with stakeholders at all stages of research, including research development, implementation, interpretation, and dissemination (Doppelt, 2017).		2	3				R		C
	Prioritize building community capacity and sustainable social infrastructure (Doppelt, 2017). Prioritize long-term community resilience through research that addresses root causes of stress and illness (Ellis and Dietz, 2017).		2	3				R		C
	Incentivize service and engagement with stakeholders and policymakers by integrating *social return* as an evaluation metric for academic promotion and funding. Offer institutional support and training for policy advocacy (Jessani et al., 2020, 2022).		2						D	C
3. Question Cultural Systems	Seek consensus through open dialogue (Mickel et al., 2023) and ‘participatory decision-making’ (Bell and Reed, 2022).	1	2	3		S	A	R	D	C
	Prevent abuse of power through the use of supervision agreements, mandatory leadership training, and arbitration of conflicts by an independent committee (Lasser et al., 2021).		2	3			A	R	D	
	Uphold shared goals over individualist objectives by practicing self-awareness and interdependence.			3		S	A	R	D	C
	Acknowledge strengths and contributions of every team member (Bennett and Gadlin, 2012).									
	Recognize and accept that knowledge-building is a *relational process*. Treat relationships and social accountabilities as outcomes of scientific progress (Tebes et al., 2014).	1	2	3		S	A	R	D	C
4. Embody Cultural Change	“Embrace humanizing epistemologies” (Rogers et al., 2024 p.489), including critical and constructivist epistemologies, as equally valid to post-positivist frameworks. Increase representation of qualitative and ideologically-situated approaches in high-impact journals (Atherton et al., 2026).		2	3	4			R		
	Openly reject practices that disempower individuals and communities on the basis of race, ethnicity, class, ability, religion, nationality, etc. Ensure academic spaces accommodate physical, auditory, and visual disabilities. Develop resources for underrepresented students (Sewell et al., 2022).		2	3	4		A	R	D	
	Critically examine cultural norms of elitism in academic institutions. Challenge expectations of productivity and prestige that reproduce these legacies (Feldon et al., 2023).			3	4			R	D	
	Use methodologies that empower and impart short- and long-term benefits for participants, communities, students, and colleagues (Kia-Keating and Juang, 2022; Smith and Ulus, 2020).	1	2	3	4			R		
	Make decisions holistically. Train research teams in acceptance, psychosocial flexibility, and dialectical thinking to facilitate generative interdisciplinary scholarship (Turner et al., 2015).	1		3	4		A	R	D	
	Protect mental and physical health of researchers by prioritizing sustainable research practices (Frith, 2020). Challenge cultural norms of competition and pressurized research performance (Smith and Ulus, 2020).	1		3	4		A	R	D	
	Recognize the socio-political context in which knowledge is situated and shared. Use “community researcher contracts” to establish open science procedures that empower rather than exploit Indigenous and other marginalized communities (Traynor and Foster, 2017).		2		4			R		C

Practices applicable for stakeholders: Students (S), Advisors (A), Researchers (R), Department (D), Community (C).

## References

[R1] Ahmed S (2012). On Being Included: Racism and Diversity in Institutional Life. New York: Duke University Press. doi: 10.2307/j.ctv1131d2g

[R2] Ahmed SM, and Palermo A-GS (2010). Community engagement in research: frameworks for education and peer review. Am. J. Public Health 100 (8), 1380–1387. doi: 10.2105/AJPH.2009.178137 20558798 PMC2901283

[R3] Akbar M, Kelly JF, Shullman SL, Jernigan M, and Faye C (2024). A historic apology: the American psychological association’s commitment to dismantling systemic racism and advancing racial equity in psychology. Am. Psychol 79 (4), 660–673. doi: 10.1037/amp0001381 39037848

[R4] American Psychological Association (2019). APA Task Force on Race and Ethnicity Guidelines in Psychology. Race and Ethnicity Guidelines in Psychology: Promoting Responsiveness and Equity. Washington, DC: American Psychological Association

[R5] Andrade MS (2006). International students in English-speaking universities: adjustment factors. J. Res. Int. Educ 5 (2), 131–154. doi: 10.1177/1475240906065589

[R6] Arcidiacono C, and Di Martino S (2016). A critical analysis of happiness and well-being. Where we stand now, where we need to go. Community Psychol. Glob. Perspect 2, 1. doi: 10.1285/i24212113v2i1p6

[R7] Atherton OE, Westberg DW, Perkins V, Lawson KM, Jeftic A, Jayawickreme E, (2026). Examining personality psychology to unpack the peer review system: towards a more diverse, inclusive, and equitable psychological science. Eur. J. Pers 40 (1), 126–152. doi: 10.1177/08902070241301629

[R8] Battiste M (2011). Reclaiming Indigenous Voice and Vision. Vancouver, BC: UBC Press.

[R9] Baumeister RF, and Leary MR (1995). The need to belong: desire for interpersonal attachments as a fundamental human motivation. Psychol. Bull 117 (3), 497–529. doi: 10.1037/0033-2909.117.3.497 7777651

[R10] Bell K, and Reed M (2022). The tree of participation: a new model for inclusive decision-making. Community Dev. J 57 (4), 595–614. doi: 10.1093/cdj/bsab018

[R11] Bennett LM, and Gadlin H (2012). Collaboration and team science: from theory to practice. J. Invest. Med 60 (5), 768–775. doi: 10.2310/JIM.0b013e318250871d PMC3652225 22525233

[R12] Bettez S (2017). Flipping the script from talking to teens about “celebrating diversity” to promoting equity through embracing vulnerability and enacting courage. Multicult. Perspect 19 (2), 90–97. doi: 10.1080/15210960.2017.1301091

[R13] Betancourt JR, Green AR, Carrillo JE, and Ananeh-Firempong O 2nd (2003). Defining cultural competence: a practical framework for addressing racial/ethnic disparities in health and health care. Public Health Rep. 118 (4), 293. doi: 10.1016/S0033-3549(04)50253-4 12815076 PMC1497553

[R14] Borshuk C (2017). Managing student self-disclosure in class settings: lessons from feminist pedagogy. J. Scholarsh. Teach. Learn 17 (1), 78–86. doi: 10.14434/josotl.v17i1.20070

[R15] Breuning M, Backstrom J, Brannon J, Gross BI, and Widmeier M (2015). Reviewer fatigue? Why scholars decline to review their peers’ work. PS Polit. Sci. Polit 48 (4), 595–600. doi: 10.1017/S1049096515000827

[R16] Buchanan NT, Perez M, Prinstein MJ, and Thurston IB (2021). Upending racism in psychological science: strategies to change how science is conducted, reported, reviewed, and disseminated. Am. Psychol 76 (7), 1097–1112. doi: 10.1037/amp0000905 34990171

[R17] Carrero Pinedo A, Caso TJ, Rivera RM, Carballea D, and Louis EF (2022). Black, Indigenous, and trainees of color stress and resilience: the role of training and education in decolonizing psychology. Psycholog. Trauma Theory Res. Pract. Policy 14 (S1), S140–S147. doi: 10.1037/tra0001187 35130018

[R18] Casad BJ, Franks JE, Garasky CE, Kittleman MM, Roesler AC, Hall DY, (2021). Gender inequality in academia: problems and solutions for women faculty in stem. J. Neurosci. Res 99 (1), 13–23. doi: 10.1002/jnr.24631 33103281

[R19] Chavez CI, and Weisinger JY (2008). Beyond diversity training: a social infusion for cultural inclusion. Hum. Resour. Manag 47 (2), 331–350. doi: 10.1002/hrm.20215

[R20] Cole EV, and Cawthon SW (2015). Self-Disclosure decisions of university students with learning disabilities. J. Postsecond. Educ. Disabil 28 (2), 163–179.

[R21] Collins PH (1986). Learning from the outsider within: the sociological significance of black feminist thought. Soc. Probl 33 (6), S14–S32. doi: 10.2307/800672

[R22] Collins SE, Clifasefi SL, Stanton J, The LEAP Advisory Board, Straits, K. J. E., Gil-Kashiwabara E, (2018). Community-Based participatory research (cbpr): towards equitable involvement of community in psychology research. Am. Psychol 73 (7), 884–898. doi: 10.1037/amp0000167 29355352 PMC6054913

[R23] Coté LE (2023). Making the case for paying undergraduate researchers in STEM disciplines. Vol. 1. doi: 10.21428/a70c814c.48bae143

[R24] Council of Graduate Schools (2008). Ph.D. Completion and Attrition: Analysis of Baseline Demographic Data from the Ph.D. Completion Project. Available online at: https://cgsnet.org/wp-content/uploads/2022/01/phd_completion_and_attrition_analysis_of_baseline_demographic_data-2.pdf (Accessed June 9, 2025).

[R25] Daniels RJ (2015). A generation at risk: young investigators and the future of the biomedical workforce. Proc. Natl. Acad. Sci. U. S. A 112 (2), 313–318. doi: 10.1073/pnas.1418761112 25561560 PMC4299207

[R26] de Cesarei A (2015). Psychological factors that foster or deter the disclosure of disability by university students. Psychol. Rep 116 (3), 665–673. doi: 10.2466/15.PR0.116k26w9 25933044

[R27] De Dreu CK, and Weingart LR (2003). Task versus relationship conflict, team performance, and team member satisfaction: a meta-analysis. J. Appl. Psychol 88 (4), 741. doi: 10.1037/0021-9010.88.4.741 12940412

[R28] Dhanani LY, Arena DF, and Bogart SM (2024). The unequal burden of DEI bans. Ind. Organ. Psychol 17 (4), 503–506. doi: 10.1017/iop.2024.44

[R29] Diener E, Pressman SD, Hunter J, and Delgadillo-Chase D (2017). If, why, and when subjective well-being influences health, and future needed research. Appl. Psychol. Health Well-Being 9, 133–167. doi: 10.1111/aphw.12090 28707767

[R30] Doppelt B (2017). Transformational Resilience: How Building Human Resilience to Climate Disruption can Safeguard Society and Increase Wellbeing. New York, NY: Routledge.

[R31] Doran JM, Kraha A, Marks LR, Ameen EJ, and El-Ghoroury NH (2016). Graduate debt in psychology: a quantitative analysis. Train. Educ. Prof. Psychol 10 (1), 2013–12013. doi: 10.1037/tep0000112

[R32] Eaton AA, Grzanka PR, Schlehofer MM, and Silka L (2021). Public psychology: introduction to the special issue. Am. Psychol 76 (8), 1209–1216. doi: 10.1037/amp0000933 35113588

[R33] Edmondson AC, and Lei Z (2014). Psychological safety: the history, renaissance, and future of an interpersonal construct. Annu. Rev. Organ. Psychol. Organ. Behav 1 (1), 23–43. doi: 10.1146/annurev-orgpsych-031413-091305

[R34] Edwards R (2026). Trusting and discomfort: a perspective on collaborating in decolonizing participatory research. Qual. Inq, 10778004251415000. doi: 10.1177/10778004251415000

[R35] Ellis WR, and Dietz WH (2017). A new framework for addressing adverse childhood and community experiences: the building community resilience model. Acad. Pediatr 17 (7), S86–S93. doi: 10.1016/j.acap.2016.12.011 28865665

[R36] Estrada M, Hernandez PR, and Schultz PW (2018). A longitudinal study of how quality mentorship and research experience integrate underrepresented minorities into stem careers. CBE–Life Sci. Educ 17 (1), ar9. doi: 10.1187/cbe.17-04-0066 29351912 PMC6007776

[R37] European Commission (2021). She Figures 2021: Gender in Research and Innovation: Statistics and Indicators. Luxembourg: Publications Office of the European Union.

[R38] Evans TM, Bira L, Gastelum JB, Weiss LT, and Vanderford NL (2018). Evidence for a mental health crisis in graduate education. Nat. Biotechnol 36 (3), 282–284. doi: 10.1038/nbt.4089 29509732

[R39] Feldman Z, and Sandoval M (2018). Metric power and the academic self: neoliberalism, knowledge and resistance in the British university. tripleC-Commun. Cap. Crit. Open Access J. Glob. Sustain. Inf. Soc 16 (1), 214–233. doi: 10.31269/triplec.v16i1.899

[R40] Feldon DF, Wofford AM, and Blaney JM (2023). “Ph.d. Pathways to the professoriate: affordances and constraints of institutional structures, individual agency, and social systems,” in Higher Education: Handbook of Theory and Research. vol 38, ed. Perna LW (Cham: Springer), 325–414. doi: 10.1007/978-3-031-06696-2_4

[R41] Finlay-Smits S, Manning S, Edwards P, Walton M, Koroheke C, and Espig M (2024). Journeys towards decolonising research practices in Aotearoa New Zealand. J. Respons. Innov 11 (1), 2347701. doi: 10.1080/23299460.2024.2347701

[R42] Flaherty C (2022). The peer-review crisis. Available online at: https://www.insidehighered.com/news/2022/06/13/peer-review-crisis-creates-problems-journals-and-scholars (Accessed May 15, 2026).

[R43] Forde AT, Crookes DM, Suglia SF, and Demmer RT (2019). The weathering hypothesis as an explanation for racial disparities in health: a systematic review. Ann. Epidemiol 31, 1–18. doi: 10.1016/j.annepidem.2019.02.011 30987864 PMC10676285

[R44] French BH, Lewis JA, Mosley DV, Adames HY, Chavez-Dueñas NY, Chen GA, (2020). Toward a psychological framework of radical healing in communities of color. Couns. Psychol 48 (1), 14–46. doi: 10.1177/0011000019843506

[R45] Friedensen RE, Bettencourt GM, and Bartlett ML (2024). Power-conscious ecosystems: understanding how power dynamics in US doctoral advising shape students’ experiences. High. Educ 87 (1), 149–164. doi: 10.1007/s10734-023-00998-x

[R46] Frith U (2020). Fast lane to slow science. Trends Cogn. Sci. (Regul. Ed.) 24 (1), 1–2. doi: 10.1016/j.tics.2019.10.007 31744772

[R47] Gewin V (2023). Postdoctoral researchers warn NIH that cost-of-living pressures are gutting the workforce. Nature. doi: 10.1038/d41586-023-02202-7 37386182

[R48] Gomez CJ, Herman AC, and Parigi P (2022). Leading countries in global science increasingly receive more citations than other countries doing similar research. Nat. Hum. Behav 6 (7), 919–929. doi: 10.1038/s41562-022-01351-5 35637294 PMC9314251

[R49] Grzanka PR, and Cole ER (2021). An argument for bad psychology: disciplinary disruption, public engagement, and social transformation. Am. Psychol 76 (8), 1334–1345. doi: 10.1037/amp0000853 35113597

[R50] Guba EG, and Lincoln YS (2005). “Paradigmatic controversies, contradictions, and emerging confluences,” in The Sage Handbook of Qualitative Research, 3rd Edn (Thousand Oaks, CA: Sage Publications Ltd.), 191–215.

[R51] Hagerty BMK, Lynch-Sauer J, Patusky KL, Bouwsema M, and Collier P (1992). Sense of belonging: a vital mental health concept. Arch. Psychiatr. Nurs 6 (3), 172–177. doi: 10.1016/0883-9417(92)90028-H 1622293

[R52] Hall KL, Vogel AL, Huang GC, Serrano KJ, Rice EL, Tsakraklides SP, (2018). The science of team science: a review of the empirical evidence and research gaps on collaboration in science. Am. Psychol 73 (4), 532–548. doi: 10.1037/amp0000319 29792466

[R53] Hamedani MG, Markus HR, Hetey RC, and Eberhardt JL (2024). We built this culture (so we can change it): seven principles for intentional culture change. Am. Psychol 79 (3), 384–402. doi: 10.1037/amp0001209 37971839

[R54] Harvey J, and Boynton K (2021). Self-Disclosure and psychological resilience: the mediating roles of self-esteem and self-compassion. Interpersona 15 (1), 90–104. doi: 10.5964/ijpr.4533

[R55] Hecker S, Bonney R, Haklay M, Hölker F, Hofer H, Goebel C, (2018). Innovation in citizen science–perspectives on science-policy advances. Citiz. Sci. Theory Pract 3 (1), 4–4. doi: 10.5334/cstp.114

[R56] Held M (2023). Decolonizing science: undoing the colonial and racist hegemony of western science. J. MultiDiscip. Eval 19 (44). doi: 10.56645/jmde.v19i44.785

[R57] Hoffer T, Grigorian K, and Hedberg E (2008). Postdoc Participation of Science, Engineering, and Health Doctorate Recipients. Arlington, VA: InfoBrief, National Science Foundation.

[R58] Hofstra B, Kulkarni VV, Munoz-Najar Galvez S, He B, Jurafsky D, and McFarland DA (2020). The diversity–innovation paradox in science. Proc. Natl. Acad. Sci. U. S. A 117 (17), 9284–9291. doi: 10.1073/pnas.1915378117 32291335 PMC7196824

[R59] Holtz P, and Odağ Ö (2020). Popper was not a positivist: why critical rationalism could be an epistemology for qualitative as well as quantitative social scientific research. Qual. Res. Psychol 17 (4), 541–564. doi: 10.1080/14780887.2018.1447622

[R60] Homer SR, Solbrig L, Djama D, Bentley A, Kearns S, and May J (2021). The researcher toolkit: a preventative, peer-support approach to postgraduate research student mental health. Stud. Grad. Postdr. Educ 12 (1), 7–25. doi: 10.1108/SGPE-06-2020-0039

[R61] Hopkins MJ, Moore BN, Jeffery JL, and Young AS (2024). Demystifying the “hidden curriculum” for minoritized graduate students. eLife 13, e94422. doi: 10.7554/eLife.94422 38470368 PMC10932539

[R62] Hordge-Freeman E (2018). “Bringing your whole self to research” the power of the researcher’s body, emotions, and identities in ethnography. Int. J. Qual. Methods 17 (1), 1609406918808862. doi: 10.1177/1609406918808862

[R63] Horta H, and Jung J (2024). The crisis of peer review: part of the evolution of science. High. Educ. Q 78 (4), e12511. doi: 10.1111/hequ.12511

[R64] Hurd F, and Singh S (2021). “Something has to change’: a collaborative journey towards academic well-being through critical reflexive practice. Manag. Learn 52 (3), 347–363. doi: 10.1177/1350507620970723

[R65] Jessani NS, Valmeekanathan A, Babcock CM, and Ling B (2020). Academic incentives for enhancing faculty engagement with decision-makers—considerations and recommendations from one school of public health. Humanit. Soc. Sci. Commun 7 (1), 148. doi: 10.1057/s41599-020-00629-1

[R66] Jessani NS, Ling B, Babcock C, Valmeekanathan A, and Holtgrave DR (2022). Advocacy, activism, and lobbying: how variations in interpretation affects ability for academia to engage with public policy. PLOS Glob. Public Health 2 (3), e0000034. doi: 10.1371/journal.pgph.0000034 36962253 PMC10021895

[R67] Joseph TD, and Hirshfield LE (2011). “Why don’t you get somebody new to do it?” race and cultural taxation in the academy. Ethn. Racial. Stud 34 (1), 121–141. doi: 10.1080/01419870.2010.496489

[R68] Juang LP (2024). “Ethnic-Racialized identities as strengths,” in Transforming Careers in Mental Health for BIPOC, 1st Edn, eds. Chang DF, and Lausell Bryant L (New York: Routledge), 13–23. doi: 10.4324/9781003309796-3

[R69] Kahu ER, and Nelson K (2018). Student engagement in the educational interface: understanding the mechanisms of student success. High. Educ. Res. Dev 37 (1), 58–71. doi: 10.1080/07294360.2017.1344197

[R70] Kazdin AE (2017). Addressing the treatment gap: a key challenge for extending evidence-based psychosocial interventions. Behav. Res. Ther 88, 7–18. doi: 10.1016/j.brat.2016.06.004 28110678

[R71] Kent BA, Holman C, Amoako E, Antonietti A, Azam JM, Ballhausen H, (2022). Recommendations for empowering early career researchers to improve research culture and practice. PLoS Biol. 20 (7), e3001680. doi: 10.1371/journal.pbio.3001680 35797414 PMC9295962

[R72] Khadakkar S, and van Nouhuys S (2024). Postdoctoral struggles in the Global South: insights from India. Sci. Nat 111 (6), 62. doi: 10.1007/s00114-024-01949-x 39527126

[R73] Kia-Keating M, and Juang LP (2022). Participatory science as a decolonizing methodology: leveraging collective knowledge from partnerships with refugee and immigrant communities. Cultur. Divers. Ethnic Minor. Psychol 28 (3), 299. doi: 10.1037/cdp0000514 35007114

[R74] Kricorian K, Seu M, Lopez D, Ureta E, and Equils O (2020). Factors influencing participation of underrepresented students in stem fields: matched mentors and mindsets. Int. J. STEM Educ 7 (1), 16. doi: 10.1186/s40594-020-00219-2

[R75] Krieger N (2020). Measures of racism, sexism, heterosexism, and gender binarism for health equity research: from structural injustice to embodied harm-an ecosocial analysis. Annu. Rev. Public Health 41, 37–62. doi: 10.1146/annurev-publhealth-040119-094017 31765272

[R76] Lala KN, and Feldman MW (2024). Genes, culture, and scientific racism. Proc. Natl. Acad. Sci. U. S. A 121 (48), e2322874121. doi: 10.1073/pnas.2322874121 39556747 PMC11621800

[R77] Lasser J, Bultema L, Jahn A, Löffler M, Minneker V, and van Scherpenberg C (2021). Power abuse and anonymous accusations in academia – perspectives from early career researchers and recommendations for improvement. Beitr. Hochschulforsch 43 (1–2), 48–61.

[R78] Lawrence L, Bishop A, and Curran J (2019). Integrated knowledge translation with public health policy makers: a scoping review. Healthc. Policy 14 (3), 55–77. doi: 10.12927/hcpol.2019.25792 31017866 PMC7008688

[R79] Lewis L (2021). Assimilation as “false consciousness”: higher education immigrant Students’ acculturation beliefs and experiences. Int. J. Intercult. Relat 83, 30–42. doi: 10.1016/j.ijintrel.2021.04.012

[R80] Linehan MM, and Wilks CR (2015). The course and evolution of dialectical behavior therapy. Am. J. Psychother 69 (2), 97–110. doi: 10.1176/appi.psychotherapy.2015.69.2.97 26160617

[R81] Liu S-NC, Brown SEV, and Sabat IE (2019). Patching the “leaky pipeline”: interventions for women of color faculty in stem academia. Arch. Sci. Psychol 7 (1), 32–39. doi: 10.1037/arc0000062

[R82] Lozada FT, Riley TN, Catherine E, and Brown DW (2022). Black emotions matter: understanding the impact of racial oppression on black youth’s emotional development: dismantling systems of racism and oppression during adolescence. J. Res. Adolesc 32 (1), 13–33. doi: 10.1111/jora.12699 34958154

[R83] Martín-Baró I (1996). Writings for a Liberation Psychology. Cambridge, MA: Harvard University Press.

[R84] McCarthy G, Song X, and Jayasuriya K (2017). The proletarianisation of academic labour in Australia. High. Educ. Res. Dev 36 (5), 1017–1030. doi: 10.1080/07294360.2016.1263936

[R85] Mickel A, Heidebrink-Bruno A, Hosseiny Marani A, Barone I, Lee O, and Baumer EP (2023). The cultural production of everyday ethics in two university STEM labs. Bull. Sci. Technol. Soc 43 (1–2), 3–17. doi: 10.1177/02704676231181030

[R86] Muhammad M, Wallerstein N, Sussman AL, Avila M, Belone L, and Duran B (2015). Reflections on researcher identity and power: the impact of positionality on community based participatory research (CBPR) processes and outcomes. Crit. Sociol. (Eugene) 41 (7–8), 1045–1063. doi: 10.1177/0896920513516025 27429512 PMC4943756

[R87] National Institutes of Health (2018). A Checklist for Communicating Science and Health Research to the Public. National Institutes of Health (NIH). Available online at: https://www.nih.gov/about-nih/what-we-do/science-health-public-trust/checklist-communicating-science-health-research-public (Accessed February 26, 2025).

[R88] Nemeth CJ (1986). Differential contributions of majority and minority influence. Psychol. Rev 93 (1), 23. doi: 10.1037/0033-295X.93.1.23

[R89] Newman SD, Andrews JO, Magwood GS, Jenkins C, Cox MJ, and Williamson DC (2011). Community advisory boards in community-based participatory research: a synthesis of best processes. Prev. Chronic. Dis 8 (3), A70. 21477510 PMC3103575

[R90] O’Connor P, Le Feuvre N, and Sümer S (2024). Cross-national variations in postdoc precarity: an inquiry into the role of career structures and research funding models. Policy Futures Educ. 22 (4), 606–624. doi: 10.1177/14782103231177483

[R91] Park CL, Kubzansky LD, Chafouleas SM, Davidson RJ, Keltner D, Parsafar P, (2023). Emotional well-being: what it is and why it matters. Affect. Sci 4 (1), 10–20. doi: 10.1007/s42761-022-00163-0 37070009 PMC10104995

[R92] Pearson H, and Boskovich L (2019). Problematizing disability disclosure in higher education: shifting towards a liberating humanizing intersectional framework. Disabil. Stud. Q 39 (1), 1. doi: 10.18061/dsq.v39i1.6001

[R93] Pfund C, House S, Spencer K, Asquith P, Carney P, Masters KS, (2013). A research mentor training curriculum for clinical and translational researchers. Clin. Transl. Sci 6 (1), 26–33. doi: 10.1111/cts.12009 23399086 PMC3572855

[R94] Pincock K, and Jones N (2020). Challenging power dynamics and eliciting marginalized adolescent voices through qualitative methods. Int. J. Qual. Methods 19, 1609406920958895. doi: 10.1177/1609406920958895

[R95] Plamondon KM, Dixon J, Brisbois B, Pereira RC, Bisung E, Elliott SJ, (2023). Turning the tide on inequity through systematic equity action-analysis. BMC Public Health 23 (1), 890. doi: 10.1186/s12889-023-15709-5 37189082 PMC10184113

[R96] Rahal R-M, Fiedler S, Adetula A, Berntsson RP-A, Dirnagl U, Feld GB, (2023). Quality research needs good working conditions. Nat. Hum. Behav 7 (2), 164–167. doi: 10.1038/s41562-022-01508-2 36755134

[R97] Roberts SO, Bareket-Shavit C, Dollins FA, Goldie PD, and Mortenson E (2020). Racial inequality in psychological research: trends of the past and recommendations for the future. Perspect. Psychol. Sci 15 (6), 1295–1309. doi: 10.1177/1745691620927709 32578504

[R98] Rodriguez Espinosa P, and Verney SP (2021). The underutilization of community-based participatory research in psychology: a systematic review. Am. J. Community. Psychol 67 (3–4), 312–326. doi: 10.1002/ajcp.12469 33165973 PMC8106689

[R99] Rogers LO, Moffitt U, McLean KC, and Syed M (2024). Research as resistance: naming and dismantling the master narrative of “good” science. Am. Psychol 79 (4), 484–496. doi: 10.1037/amp0001246 39037835

[R100] Samaras G (2025). Battleground Europe: the rise of anti-woke movements and their threat to democracy. Front. Polit. Sci 7, 1568816. doi: 10.3389/fpos.2025.1568816

[R101] Segev E, and Hochman Y (2023). Teaching note—the hidden key: opening the door to self-disclosure in social work education. J. Soc. Work. Educ 59 (4), 1258–1264. doi: 10.1080/10437797.2022.2039822

[R102] Settles IH, Jones MK, Buchanan NT, and Dotson K (2021). Epistemic exclusion: scholar (ly) devaluation that marginalizes faculty of color. J. Divers. High. Educ 14 (4), 493. doi: 10.1037/dhe0000174

[R103] Sewell A, Kennett A, and Pugh V (2022). Universal design for learning as a theory of inclusive practice for use by educational psychologists. Educ. Psychol. Pract 38 (4), 364–378. doi: 10.1080/02667363.2022.2111677

[R104] Shahram SZ (2023). Five ways “health scholars” are complicit in upholding health inequities, and how to stop. Int. J. Equity Health 22 (1), 15. doi: 10.1186/s12939-022-01763-9 36658523 PMC9851581

[R105] Shin JC, Postiglione GA, and Ho KC (2018). Challenges for doctoral education in east Asia: a global and comparative perspective. Asia Pac. Educ. Rev 19 (2), 141–155. doi: 10.1007/s12564-018-9527-8

[R106] Smith C, and Ulus E (2020). Who cares for academics? We need to talk about emotional well-being including what we avoid and intellectualise through macro-discourses. Organization 27 (6), 840–857. doi: 10.1177/1350508419867201

[R107] Spoon K, LaBerge N, Wapman KH, Zhang S, Morgan AC, Galesic M, (2023). Gender and retention patterns among U.S. faculty. Sci. Adv 9 (42), eadi2205. doi: 10.1126/sciadv.adi2205 37862417 PMC10588949

[R108] Steck H (2003). Corporatization of the university: seeking conceptual clarity. Ann. Am. Acad. Pol. Soc. Sci 585 (1), 66–83. doi: 10.1177/0002716202238567

[R109] Stephens NM, Fryberg SA, Markus HR, Johnson CS, and Covarrubias R (2012). Unseen disadvantage: how American Universities’ focus on independence undermines the academic performance of first-generation college students. J. Pers. Soc. Psychol 102 (6), 1178–1197. doi: 10.1037/a0027143 22390227

[R110] Tebes JK, Thai ND, and Matlin SL (2014). Twenty-first century science as a relational process: from Eureka! to team science and a place for community psychology. Am. J. Community Psychol 53 (3), 475–490. doi: 10.1007/s10464-014-9625-7 24496718 PMC4076783

[R111] Teng MY, Brown M-L, Jarus T, and Yvonne Bulk L (2020). How does a sense of belonging develop in postsecondary? A conceptual belonging in academia model (bam) from sighted perspectives. Res. Educ 108 (1), 80–103. doi: 10.1177/0034523719882455

[R112] Traynor C, and Foster L (2017). Open and Collaborative Science in Development Network (OCSDNet). Principles and Practice in Open Science: Addressing Power and Inequality Through “Situated Openness.” Available online at: https://ocsdnet.org/principles-and-practice-in-open-science-addressing-power-and-inequality-through-situated-openness/ (Accessed February 06, 2025).

[R113] Turner VK, Benessaiah K, Warren S, and Iwaniec D (2015). Essential tensions in interdisciplinary scholarship: navigating challenges in affect, epistemologies, and structure in environment–society research centers. High. Educ 70 (4), 649–665. doi: 10.1007/s10734-015-9859-9

[R114] Voith LA, Hamler T, Francis MW, Lee H, and Korsch-Williams A (2020). Using a trauma-informed, socially just research framework with marginalized populations: practices and barriers to implementation. Soc. Work. Res 44 (3), 169–181. doi: 10.1093/swr/svaa013

[R115] Wang J, Cheng GH-L, Chen T, and Leung K (2019). Team creativity/innovation in culturally diverse teams: a meta-analysis. J. Organ. Behav 40 (6), 693–708. doi: 10.1002/job.2362

[R116] Warner LR (2008). A best practices guide to intersectional approaches in psychological research. Sex. Roles 59 (5–6), 454–463. doi: 10.1007/s11199-008-9504-5

[R117] Welch A, and Li J (2021). Measuring up in Higher Education: How University Rankings and League Tables are Re-Shaping Knowledge Production in the Global Era. Singapore: Springer Nature.

[R118] Wilcox MM, Barbaro-Kukade L, Pietrantonio KR, Franks DN, and Davis BL (2021). It takes money to make money: inequity in psychology graduate student borrowing and financial stressors. Train. Educ. Prof. Psychol 15 (1), 2–17. doi: 10.1037/tep0000294

[R119] Williamson HJ, Chief C, Jiménez D, Begay A, Milner TF, Sullivan S, (2020). Voices of community partners: perspectives gained from conversations of community-based participatory research experiences. Int. J. Environ. Res. Public Health 17 (14), 5245. doi: 10.3390/ijerph17145245 32708111 PMC7400085

[R120] Wilson-Mendenhall CD, and Holmes KJ (2023). Lab meets world: the case for use-inspired basic research in affective science. Affect. Sci 4 (3), 591–599. doi: 10.1007/s42761-023-00200-6 37744977 PMC10514004

[R121] Xu H, Bu Y, Liu M, Zhang C, Sun M, Zhang Y, (2022). Team power dynamics and team impact: new perspectives on scientific collaboration using career age as a proxy for team power. J. Assoc. Inf. Sci. Technol 73 (10), 1489–1505. doi: 10.1002/asi.24653

[R122] Xu H, Liu M, Bu Y, Sun S, Zhang Y, Zhang C, (2024). The impact of heterogeneous shared leadership in scientific teams. Inf. Process. Manag 61 (1), 103542. doi: 10.1016/j.ipm.2023.103542

