# Supplementary material for: Embodying well-being in research: key principles and praxis
Source: Front Educ (Lausanne). Author manuscript; Available in PMC 2026 Jul 17. (PMC13375101; doi:10.3389/feduc.2026.1836519)
Supplement: Figure S1 [file NIHMS2192174-supplement-Figure_S1.docx]

# **Supplemental Materials**


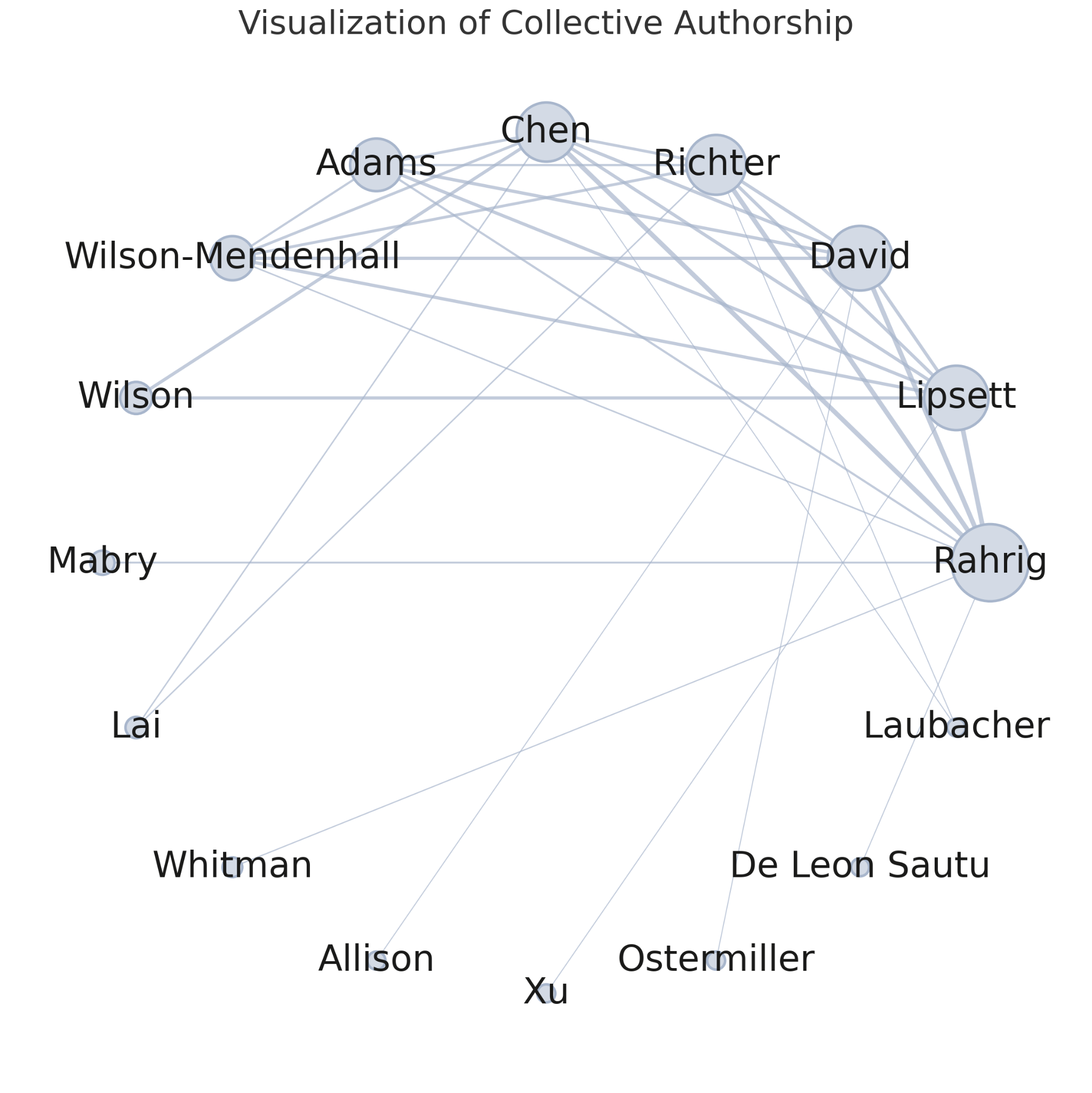


**Figure S1. Visualizing the Collective Authorship: A Hybrid Representation.** To emphasize our team science approach, we developed a visual representation of authorship that highlights collaborative relationships. Node size reflects the total points each author earned using a contribution-based system adapted from Chafouleas et al. (2020). Edges represent interactions between authors throughout manuscript preparation.
